# Dysfunction of Tregs contributes to FGR pathogenesis via regulating Smads signalling pathway

**DOI:** 10.1111/jcmm.15059

**Published:** 2020-02-14

**Authors:** Yunzhao Xu, Min Su, Ziheng Wang, Qinqin Liu, Xiangyu Xu, Shuting Gu, Weidong Pan, Wenliang Ge

**Affiliations:** ^1^ Department of Obstetrics Affiliated Hospital of Nantong University Nantong Jiangsu China; ^2^ Department of Obstetrics and Gynecology Nantong University Nantong Jiangsu China; ^3^ Department of Clinical Biobank Affiliated Hospital of Nantong University Nantong Jiangsu China; ^4^ Department of Pediatric Surgery Affiliated Hospital of Nantong University Nantong Jiangsu China

**Keywords:** fetal growth restriction, Foxp3, Smads signaling pathway, T regulatory cells

## Abstract

Fetal growth restriction (FGR) is ranked number two of most common complication of abnormal pregnancy worldwide. The pathogenesis of FGR is complicated due to multiple aetiologies and the exact mechanism for FGR development is currently unknown. T regulatory cells (Tregs) are proven to play central roles in the maintenance of normal pregnancy. Peripheral blood samples of 102 pregnant human were collected analysed using flow cytometry to identify Tregs. We found that reduced Tregs and down‐regulation of Foxp3 were observed in peripheral blood of FGR patients. In FGR mouse model, we have found that Tregs were not only reduced in spleen but also in placenta. In *vitro*, Foxp3 and its transcription regulatory signalling molecules, including P‐Smad2, P‐Smad3 and Smad4, were diminished as well. Inhibition on Foxp3 expression was partially reversed by overexpression of Smad2 and Smad4. In FGR patients, Western blot results revealed that Foxp3, P‐Smad2, P‐Smad3 and Smad4 expression was inhibited in placenta. Our preliminary result suggests that maternal‐foetal immune tolerance mediated by Tregs plays an essential role in the development of FGR. The inhibited expression of Foxp3 and down‐regulated Smad2/Smad3/Smad4 signalling pathway were involved in the FGR pathogenesis. Targeting maternal‐foetal immune tolerance through Tregs might represent a novel therapeutic option for FGR.

## 1 INTRODUCTION

Used to be called intrauterine growth restriction (IUGR), FGR is the second most major cause of perinatal mortality currently, responsible for 30% of perinatal death worldwide.^1^ It is not only a common cause for perinatal morbidity and accompanying multiple neonatal complications,^2^ but it also severely affects the physical and mental development of the newborns. Its effect can even extend to childhood, puberty and adulthood, with association of cardiovascular, neurological and metabolic diseases. The incidence of FGR worldwide is 2.75%‐15.53%^3^ of all pregnancies, and 8.7% in China where 1,800,000 new FGR cases occur each year. Clinically, FGR is defined as the foetal weight below the 10th percentile on gestational age. The pathogenesis of FGR is complicated. Recent studies suggest that placenta malfunction,^4^ uterus infection,^5^ genetic factors and race,^6^ as well as environmental pollutions,^7^ are all possible causes of FGR. However, the exact mechanism for FGR development is unclear.

Several lines of evidence suggest that maternal immune tolerance to the semiallogenic foetus plays important role in a successful pregnancy and dysregulation of this immune balance is closely related to the development of FGR.^8^ Fas ligand (FasL) can bind to its receptor (Fas) on activated immune cells, leading to immune cell apoptosis, which limits the host immune response. During normal pregnancy, maternal immune tolerance is essential for the proper development of the foetus. One mechanism suggests that foetal trophoblast cells express FasL, which subsequently induces apoptosis of activated lymphocytes. Karthikeyan et al indicated that FasL induced by decidual cells was reduced remarkably in FGR pregnancy compared to normal pregnancy, that peripheral blood level of FasL was significantly lower in FGR patients than in normal controls, and that low level FasL was correlated with lower placenta and newborn weights.^9^ Placenta expression of human histocompatibility antigen‐G mRNA which inhibits natural killer cells and supports survival of semiallogenic foetus was significantly decreased in FGR patients compared to normal controls.^10^ Finally, abnormal maternal inflammation can lead to FGR via increased secretion of tumour necrosis factor‐a (TNF‐a).^11^

T regulatory cells (Tregs) are a special subtype of T cells, important for immune regulation. They are marked by CD4, CD25 and Foxp3. Both peripheral blood Tregs and spleen Tregs take a great part in immune homeostasis and immune tolerance during pregnancy. During normal pregnancy, Tregs are increased at the early phase of pregnancy and maintained at constant level until the end of pregnancy.^12^ Tregs induce immune suppression via secreting several suppressive cytokines (IL‐10, TGF‐β), or through direct cell‐cell interaction. In the abortion‐prone mice, adoptive transfer of pregnancy‐induced Tregs can ameliorate the foetal absorption.^13^ Female mice lacking conserved non‐coding DNA sequence 1 (CNS1) have increased rate of miscarriage due to inability to induce Tregs.^14^ It is established that Tregs are involved in miscarriage of unknown cause, but is not known whether Tregs play a role in FGR as well.

In our study, the diminished proportion of Tregs and reduced Foxp3 expression were observed in peripheral blood of FGR patients. Tregs were not only reduced in spleen but also in placenta in FGR mouse model. In *vitro*, the expression of Foxp3 and its transcription regulatory signalling molecules, including P‐Smad2, P‐Smad3 and Smad4, were diminished. Overexpression of Smad2 and Smad4 could partially abrogate the inhibitory effect on Foxp3 expression. Our findings indicate that dysfunction of Tregs may be involved in FGR pathogenesis via regulating Smad2/Smad3/Smad4 signalling pathway.

## 2 MATERIAL AND METHODS

### 2.1 Selection of patients

We conducted a prospective study between December 2011 and June 2019 in Nantong University Affiliated Hospital. Informed consent was acquired from patients who visited the obstetrics department prior to sample collection. Pregnancy starting date was determined based on ultrasound measurements between 8‐12 gestational weeks. Pregnancies with foetuses having congenital or chromosomal abnormalities or multiple pregnancies were excluded. According to the American Academy of Family Physicians (AAFP) growth curves,^15^ patients were divided into the following groups: 1. Term (＞37 weeks) and appropriate for gestational age (AGA ＞ 10th percentile); 2. Term and small for gestational age (SGA ≤ 10th percentile). Both maternal serum and placentas were gathered during delivery. Demographics information about the patients was recorded in detail as following (Table 1).

**Table 1:** Demographics of the study population

	Term AGA	Term SGA	*P* value
Sample size(n)	62	40	
Gestational age	39 (37‐41)	39 (37‐41)	.8216
Foetal weight(g)	3511 (2550‐4300)	2128 (1350‐2500)	＜.05
Gender(%male)	46.8%	42.5%	.5978
Mode of Delivery(%Vaginal)	53%	27.5%	＜.05
Apgar at 1 min	9.8(6‐10)	9(3‐10)	＜.05
Apgar at 5 min	9.9(8‐10)	9.7(5‐10)	＜.05

Note

Continuous data are reported as median and classified as percentages.

Chi‐square test conducted for categorical variables and Kruskal–Wallis ANOVA for continuous variables. *P* ＜ .05 was defined as significance.

### 2.2 Ethics Statement

The prospective study protocol and all procedures were approved by the Ethics Committee of Nantong University Affiliated Hospital. Animal experiments were performed based on the permission of Animal Care Committee of Nantong University (no. 20170304‐001) according to the recommendations from the Care and Use of Laboratory Animals (Ministry of Science and Technology of China, 2016).

### 2.3 Mouse Experiments

IFNAR^−/−^ mice on the B6 background were kept under specific pathogen‐free conditions. Considering the fertilization day, the presence of a vaginal plug, indicating embryonic day (E) 0.5, was assessed daily. Zika virus (ZIKV) 1 × 10^5^ PFU infected pregnant dams intraperitoneally at E10.5. All the infected mice were killed with their spleen, placentas and foetuses harvested on E18.5. The measurement of foetal size is the crown‐rump length multiplied by occipito‐frontal diameter of the head.^16^

### 2.4 Cells and Viruses

EL4 cell line (T cell thymoma cell line), with T cell properties which utilized exploring T cells transcriptional regulation widely, was obtained from Cell Resource Centre of Shanghai Life Science Institute. EL4 cells and Vero cells were maintained in DMEM medium containing 10% FBS. ZIKV stock was generated from Vero cells and ZIKV titrations were determined by titration.

### 2.5 RNA Isolation and qRT‐PCR

Total RNA was isolated utilizing TRIzol reagent (Thermo Fisher Scientific), and cDNA was synthesized utilizing the iScript cDNA synthesis kit (Bio‐Rad) according to the instructions. Primers (Foxp3 forward primer: GTGGCCCGGATGTGAGAAG; Foxp3 reverse primer GGAGCCCTTGTCGGATGATG; Smad4 forward primer: ACACCAACAAGTAACGATGCC; Smad4 reverse primer: GCAAAGGTTTCACTTTCCCCA.) were designed using the DNA MAN software. Quantitative RT‐PCR (qRT‐PCR) was performed to measure the abundance of target genes by using a Bio‐Rad CFX96 PCR instrument. Gene mRNA relative expression was quantified using the 2^−ΔΔCT^ method.

### 2.6 Flow cytometric analysis

Maternal placenta samples were gathered, and single‐cell suspensions were prepared according to Tang et al^17^ The methods of flow cytometry staining were performed according to published criteria.^18^

In brief, isolated mononuclear cells were stained by FITC‐CD4, APC‐CD25 and PE‐Foxp3 which acquired from eBioscience (San Diego, CA, USA). Further analysis about staining was performed using FlowJo software (version 10, Tree Star).

### 2.7 Isolation of Tregs and Proliferation assay

CD4^+^CD25^+^ T cells were isolated from the splenocytes according to the manufacturer's instructions. Tregs, whose purity was between 96% and 98%, was used to do subsequent experiments. CD4^+^CD25^−^ T cells isolated from naive mice were stimulated with 1 µg/ml anti‐CD3 mAb in the presence of CD4^+^CD25^+^ T cells isolated from PBS‐injected and ZIKV‐injected pregnant mice and cultured for 72 hours. ELISA 5‐bromo‐2‐deoxyuridine (BrdU) kit (Roche Applied Science, Mannheim, Germany) was utilized to assay the proliferation.

### 2.8 Cell electroporation

The methods of pcDNA3.1‐Smad2 or pcDNA3.1‐Smad4 construction were performed according to published criteria.^18^ pcDNA3.1‐Smad2 or pcDNA3.1‐Smad4 was transferred into EL4 cells using the Amaxa Nucleofector System. Subsequently, EL4 cells were infected with ZIKV at a MOI of 2 for 2 hours.

### 2.9 Western Blot

Proteins were separated and transferred to PVDF membranes at 300 mA for 2 hours. After blocking, the membranes were then incubated with anti‐P‐Smad2, P‐Smad3, Smad2, Smad3, Smad4 (Cell Signaling Technology, Danvers, MA, USA) and Foxp3 (Abcam, Cambridge, MA, USA) antibodies, respectively, overnight at 4℃. The goat anti‐rabbit IgG secondary antibodies were incubated for the membranes at room temperature for 2 hours. Target protein bands were visualized by enhanced chemiluminescence kit (ECL, Roche Diagnostics), normalized to GAPDH and then determined by Image J (National Institutes of Health, Bethesda, MD).

### 2.10 Immunohistochemistry analysis

Placenta tissue sections from normal pregnancy and FGR patients were deparaffinized and rehydrated through graded alcohols. Endogenous peroxidase activity was blocked by incubation in 3% H_2_O_2_. Antigen retrieval was carried out with 0.01 M citrate buffer pH 6.0 and microwave induction heat. Immunostaining was performed using anti‐Foxp3 (Abcam, Cambridge, USA) and anti‐Smad4 (Cell Signaling Technology, MA, USA) as the primary antibody and then incubated with the secondary antibody (SantaCruz, CA, USA). Nuclei were visualized with haematoxylin on each slide, which imaged by Olympus BX40 with CellSens Dimension software.

### 2.11 Data Statistical Analysis

All data statistical analyses were calculated with SPSS20 statistic software and STATA 12.0. Data were showed as mean ± SD. *t* test was performed to identify the differences between individual treatment and control groups. The results of multiple group comparisons were analysed with ANOVA.

## 3 RESULTS

### 3.1 FGR pregnancy was associated with reduced number of Tregs and down‐regulation of Foxp3 in peripheral blood

**Figure 1:**
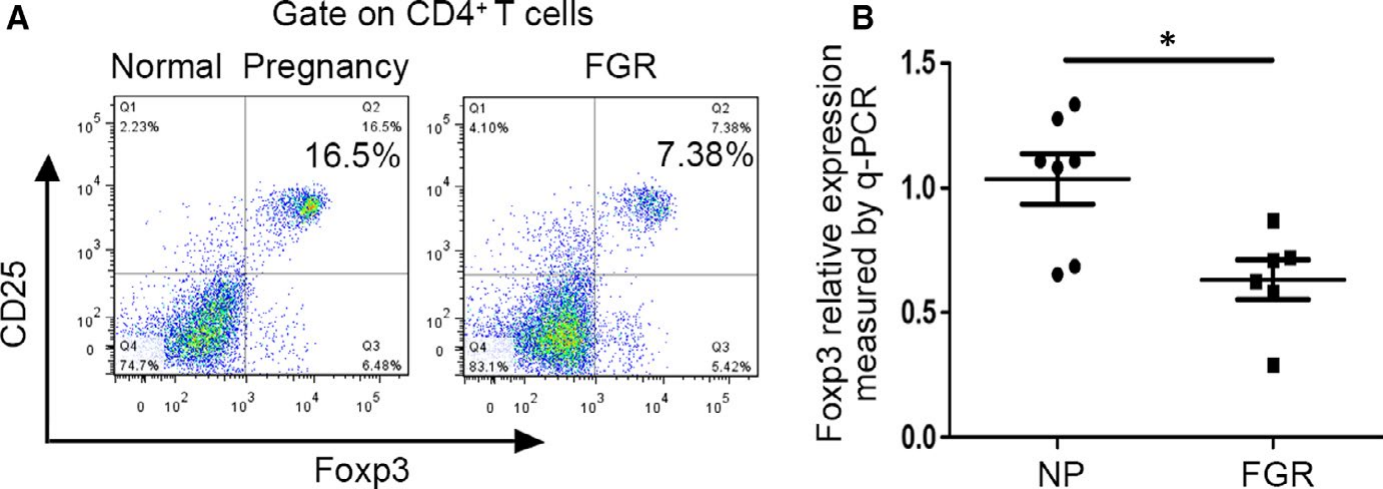
Proportion of Treg cells and Foxp3 expression in peripheral blood samples from FGR pregnancy. A, flow cytometry analysis of Treg cells percentage among CD4^+^ T cells in peripheral blood samples from normal (NP) and FGR pregnancy. B, Foxp3 mRNA expression in peripheral blood samples from normal (NP) and FGR pregnancy

Maternal Tregs play a dominant role in maintaining immune tolerance during pregnancy.^19^ During normal pregnancy, maternal Tregs cells with foetal specificity accumulate, while the number of such Treg cells are reduced during unexplained infertility, miscarriage and preeclampsia.^20^ To determine whether Tregs play a role in FGR pregnancy as well, we collected peripheral blood samples from 62 normal pregnancy and 40 from FGR pregnancy for flow cytometer analysis. The proportion of CD4^+^/CD25^+^/Foxp3^+^ lymphocytes among CD4^+^ lymphocytes in FGR pregnancy (7.38%) was significantly lower than in normal pregnancy (16.5%) (*P* = .011) (Figure 1A). In addition, Foxp3 expression in peripheral blood was significantly lower in FGR pregnancy than in normal pregnancy (Figure 1B). These data suggest that the deficit in Treg cell number may be involved in FGR pathogenesis.

### 3.2 Diminished number and reduced function of Tregs were observed in FGR mouse model

**Figure 2:**
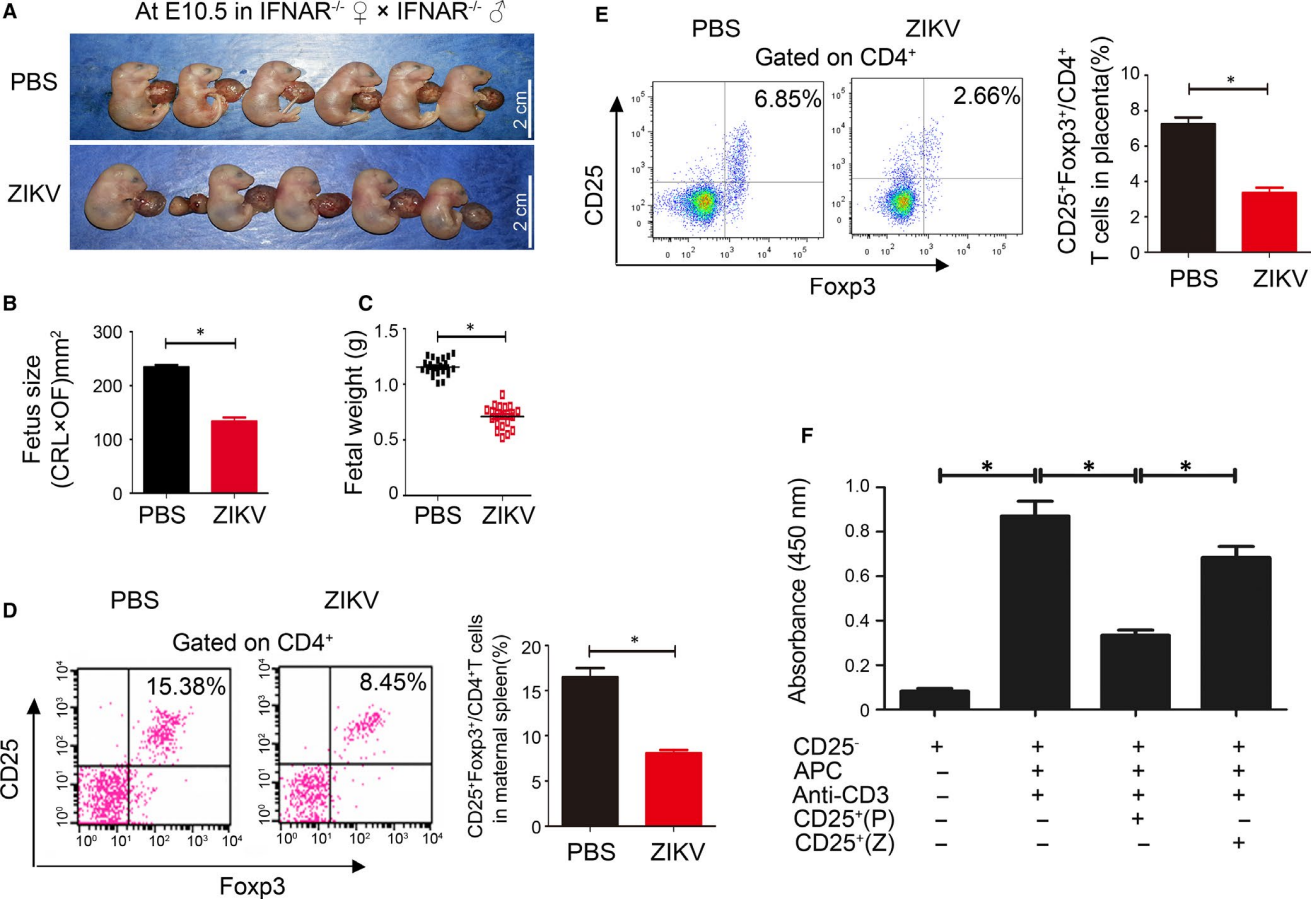
Proportion of Treg cells among CD4^+^ T cells in mouse FGR model. A, Morphological changes of foetal mice in FGR model. B, Foetal mouse size was calculated by multiplying crown‐rump length with head circumference. C, Foetal mouse weight was significantly lower in FGR model. D, The proportion of Treg cells among CD4^+^ T cells was significantly lower in spleen from FGR model. E, The proportion of Treg cells among CD4^+^ T cells was significantly lower in placenta from FGR model. F, Responder CD4^+^CD25^－^ T cells (1 × 10^5/^well) from naive mice were cultured with naive, irradiated APC (1 × 10^5^/well) and CD4^+^CD25^+^T cells (5 × 10^4^/well) harvested from pregnant mice injected with PBS (P), and ZIKV (Z), respectively. The data shown were performed in triplicate and were representative of three independent experiments. *P < .05

To further explore the correlation between Tregs dysfunction and FGR pathogenesis, we establish FGR mouse model by ZIKV infection. Mice pregnant at E10.5 were injected with ZIKV and followed until E18.5. ZIKV‐infected mice had significantly lower bodyweight starting at E15.5. Further, we showed that both size and weight of foetuses from ZIKV‐infected mice were significantly reduced compared to normal mice (Figure 2A‐2C). Thus, we successfully established FGR mouse model. Then, we determined the number of Tregs in the spleen of FGR pregnancy mice by flow cytometer. As shown in Figure 2D, the percentage of CD4^+^/CD25^+^/Foxp3^+^ cells in the spleen of FGR mice (8.45%) was significantly lower than in the spleen of control group (15.38%). The percentage of CD4^+^/CD25^+^/Foxp3^+^ cells in the placenta of FGR mice (2.66%) was also significantly lower than in the placenta of control group (6.85%) (Figure 2E). To further assess the function of Tregs, we isolated the primary Tregs (CD4^+^CD25^+^ T cells) from FGR mouse model. ZIKV infection resulted in the attenuation of the inhibitory capacity of CD4^+^CD25^+^ T cells (Figure 2F). These data indicated the diminished number and reduced function of Tregs may be correlated with FGR pathogenesis.

### 3.3 ZIKV infection lowered Foxp3 expression in EL‐4 cells

**Figure 3:**
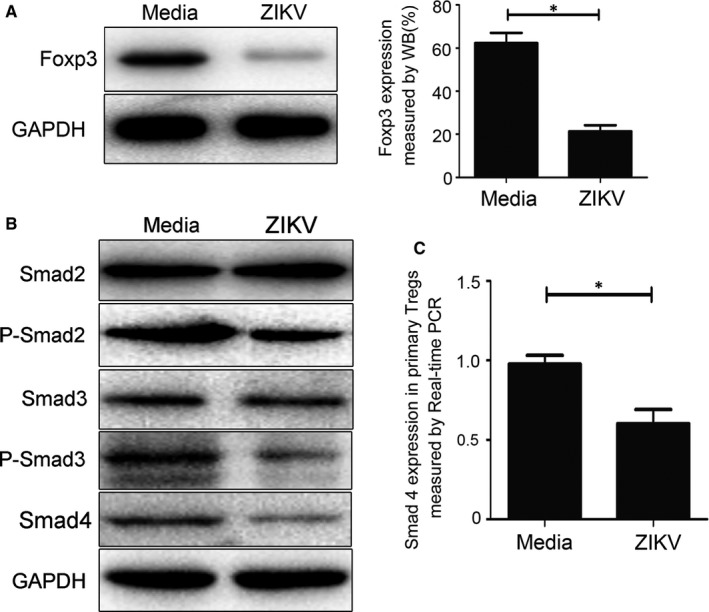
Expression of Foxp3 and Smads in EL4 cells or primary Tregs. A, Western blot analysis of Foxp3 protein expression in ZIKV‐infected EL4 cells. B, Western blot analysis of Smad2, Smad3 and Smad4 protein expression as well as phosphorylation of Smad2 and Smad3 proteins in ZIKV‐infected EL4 cells. C, Real‐time PCR analysis of Smad4 mRNA expression in ZIKV‐infected primary Tregs.*P < .05. *P < .05

To determine the mechanism of how ZIKV reduced Tregs, we directly infected murine lymphoblast cell line EL‐4. We showed that ZIKV infection could directly down‐regulate Foxp3 protein expression (Figure 3A). As Smad signalling pathway is directly involved in Foxp3 expression induction and maintenance,^21^ we determined the expression of Smad2, Smad3 and Smad4, as well as P‐Smad2 and P‐Smad3. As shown in Figure 3B, ZIKV infection did not affect Smad2 and Smad3 expression, but significantly down‐regulated Smad4 expression as well as P‐Smad2 and P‐Smad3 (*P* < .05). These data suggest that in ZIKV‐induced FGR animal model, Smad signalling pathway might directly participate in FGR pathogenesis.

### 3.4 Foxp3 expression was inhibited through Smad2/3/4 signalling pathway

**Figure 4:**
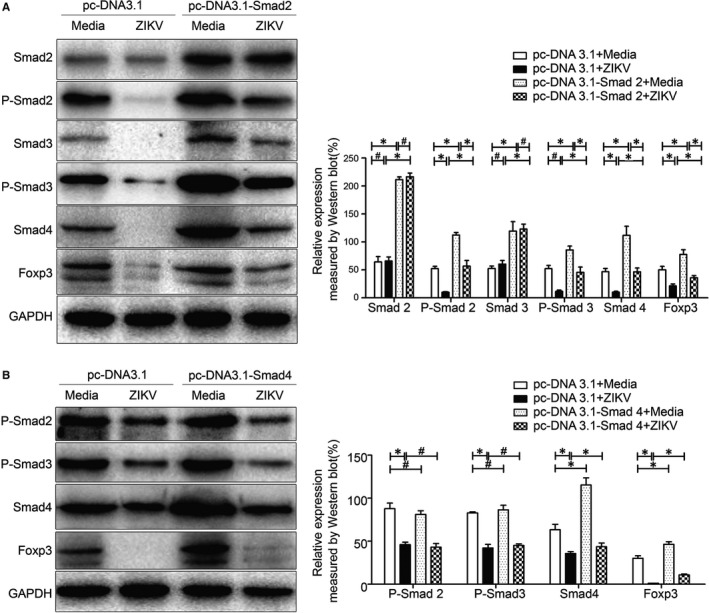
Influence on Foxp3 and Smads expression by overexpression of Smads. A, Western blot analysis of Foxp3, Smad2, Smad3 and Smad4 protein expression as well as phosphorylation of Smad2 and Smad 3 in Smad2 overexpressing EL4 cells (pc‐DNA3.1‐Smad2). B, Western blot analysis of Foxp3 and Smad4 protein expression as well as phosphorylation of Smad2 and Smad 3 in Smad4 ‐overexpressed EL4 cells (pc‐DNA3.1‐Smad4). *P < .05, ^#^ *P* > .05

To further elucidate the mechanism of Smad2/3/4 signalling pathway in FGR pathogenesis, we overexpressed Smad2 and Smad4 and determined their effects on Smad2 and Smad4 expression as well as their downstream genes. We showed that overexpression of Smad2 significantly enhanced P‐Smad2 as well as Foxp3 expression level, and ZIKV infection still significantly reduced level of P‐Smad2, P‐Smad3, Smad4 and Foxp3. However, compared to ZIKV infection in mock transfection (no Smad2 overexpression), overexpression of Smad2 significantly increased level of Smad3, P‐Smad3, Smad4 and Foxp3 (Figure 4A). Overexpression of Smad4 exerted no influence on P‐Smad2 and P‐Smad3 expression, but significantly increased Smad4 and Foxp3 expression, ZIKV infection still significantly reduced P‐Smad2, P‐Smad3, Smad4 and Foxp3 expression. However, compared to ZIKV infection in mock transfection (no Smad4 overexpression), overexpression of Smad4 remarkably up‐regulated Smad4 and Foxp3 expression (Figure 4B). Our data suggested that ZIKV down‐regulated Foxp3 expression through Smad2/3/4 signalling pathway.

### 3.5 Smad2/3/4 signalling pathway participated in FGR pathogenesis

**Figure 5:**
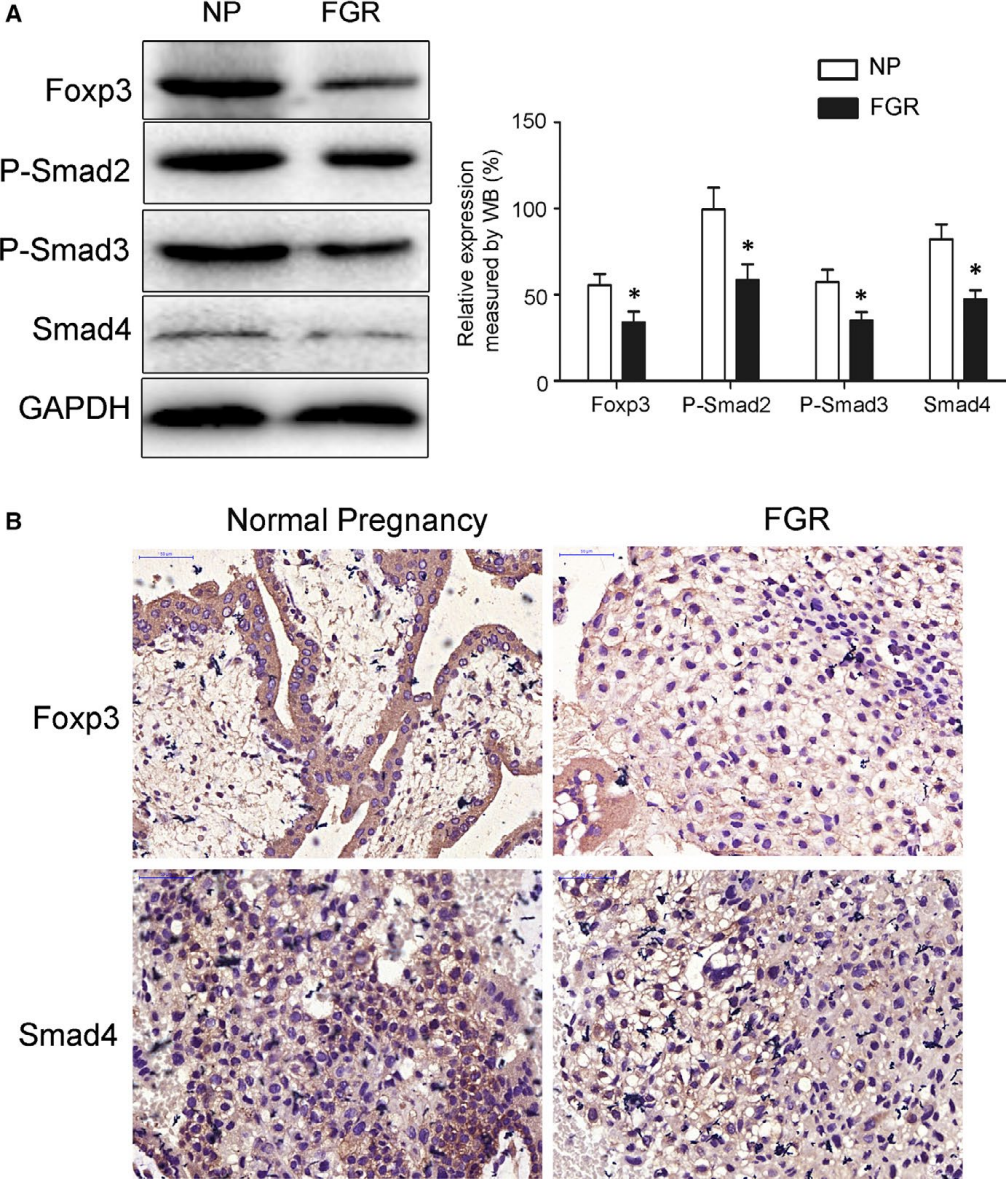
Foxp3 and Smad4 protein expression in placenta from FGR pregnancy. A, Western blot analysis of Foxp3 and Smad4 protein expression as well as phosphorylation of Smad2 and Smad 3 protein in placenta from normal and FGR pregnancy. B, Immunohistochemistry analysis of Foxp3 and Smad4 protein expression in placenta from normal and FGR pregnancy. *P < .05

In order to further confirm the involvement of Smad2/3/4 signalling pathway in FGR pathogenesis, we determined the expression levels of relevant proteins in the placenta of FGR patient. Analysis of Western blot identified that Foxp3 was remarkably down‐regulated, as well as P‐Smad2, P‐Smad3 and Smad4 (Figure 5A). Immunohistochemistry analysis further confirmed down‐regulation of Foxp3 and Smad4 in the placenta from FRG patients (Figure 5B). Our data suggested that Smad2/3/4 signalling pathway participated in FGR pathogenesis.

## 4 DISCUSSION

We have found that Treg cells reduced in maternal peripheral blood from FGR patients. To further investigate the role of Tregs in FGR, we successfully established the FGR mouse model. Tregs were diminished in both the spleen and the placenta in this mouse model. In *vitro*, we found that ZIKV infection reduced Foxp3 expression in EL‐4 cells, as well as Smad4 in addition to the reduced phosphorylation of Smad2 and Smad3. Inhibition on Foxp3 expression was partially reversed by Smad2 and Smad4 overexpression. The reduced level of P‐Smad2, P‐Smad3, and Smad4 was observed in the FGR placentas as well. We concluded that dysfunction of Tregs was involved in the FGR pathogenesis via Smad2/Smad3/Smad4 signalling pathway.

Consistent with our findings in FGR, several studies have shown that the deficit in Tregs number was associated with several pregnancy complications. Necrotizing enterocolitis (NEC) is a frequent complication in FGR newborns. The proportion of Tregs among CD4^+^ T cells was only 2.9% in NEC newborns compared to 6.6% in normal newborns without NEC.^22^ Tregs numbers were significantly lower in FGR cord blood than in normal cord blood, accompanied with suppressed expression of Foxp3.^15^ Preeclampsia is the major cause for pregnant women and perinatal death, and FGR is the most common consequence of preeclampsia.^23^ In rat preeclampsia‐induced FGR model, the total numbers of CD3^+^, CD4^+^ and CD8^+^ T cells in FGR placenta were comparable to control placenta. However, the proportion of Tregs among CD4^+^ T cells was significantly decreased in FGR placenta. When preeclampsia was alleviated with CD28 monoclonal antibody treatment (JJ316) in rat preeclampsia model, the number of Tregs in peripheral blood and the weight of foetal rats were increased. This suggests that increased Tregs could reduce preeclampsia‐induced FGR.^24^

Recent studies suggest that additional immune suppressive cells are present to establish immunological tolerance during pregnancy, including regulatory B cells (Bregs), myeloid derived suppressor cells (MDSCs) and natural killer T cells (NK T cells). Negative regulation of Bregs may be attributed to their capacity to produce anti‐inflammatory cytokines, especially IL‐10.^25^ Bregs hamper antigen presentation and inhibit costimulatory molecules, including CD80, CD86. And Bregs suppress the activation of and cytokine released by T cells in immune responses and induce Tregs differentiation.^26^ The level of MDSC is raised in the pregnancy and the neonate, which may aid to dampen immune responses to maintain immunological tolerance during pregnancy. Myeloid derived suppressor cells immunosuppression results in the down‐regulation of T cell receptor (TCR) and cell proliferation via reduction of arginine levels.^27^ Enhanced NKT cells in recurrent abortion or implantation failure lead to pregnancy loss can be ameliorated with intravenous immunoglobulin (IVIG) treatment, and achieve successful pregnancy.^28^ Future studies should investigate their roles in FGR as well as interaction among these different types of immune suppressive cells.

Foxp3 is a specific transcription factor of Tregs, which regulate Tregs differentiation and sustain Treg function. Foxp3 overexpression in CD4^+^ T cells repressed cytokine gene expression, including IL‐2, IFN‐g, IL‐4, and IL‐17.^29^ Non‐functional fusion protein of Foxp3 in mice with a Foxp3 allele made CD4^+^ T cells lack suppressor function, suggesting that the expression of functional Foxp3 protein are required for inhibitory function of Tregs.^30^ Mice lacking Foxp3 expression have deficits in Tregs and develop autoimmune disease similar to IL‐2R^‐/‐^ mice. Foxp3 deficiency is also associated with preeclampsia and unexplained miscarriage. Toldi G revealed that the proportion of CD4^+^ CD25^+^ Foxp3^+^ Tregs was lower in preeclampsia patients, which have comparable levels to the non‐pregnant women.^31^ In pathogen‐induced miscarriage animal model, Foxp3 expression is inhibited in peripheral blood, inguinal lymph nodes, and spleen as well as at the maternal‐foetal interface.^32^ In spontaneous abortion patients, Foxp3 expression are dramatically reduced in CD4^+^CD25^+^ T cells in peripheral blood.^33^ Our study indicated that Foxp3 expression is suppressed in peripheral blood as well as placenta from FGR patients. Consistent with these results, Foxp3 deficit might be involved in FGR pathogenesis.

Several signalling pathways are implicated in Foxp3 regulation, including TGF‐β/Smads, IL‐2R/STATs and PI3K/Akt/mTOR. Historically, the TGF‐β/Smads pathway has been regarded as one of the most considerable signal pathways for regulating Foxp3 expression and sustaining Treg function.^21^ Naïve T cells could convert to Foxp3^+^ Tregs in *vivo*, upon exposure to TCR and TGF‐β1 stimulations. It is reported that Smad2 and Smad3 serve as key regulators for TGF‐β–mediated induction of Foxp3 transcription and inhibition of Th1 development.^34^ Smad2/Smad3‐double knockout mice, lead to fatal inflammatory diseases accompanied with diminished Foxp3 expression in CD4^+^T cells and enhanced IFN‐gamma production at the periphery. Deletion of Smad3 remarkably resulted in a decline in TGF‐β1‐induced Tregs generation, indicating TGF‐β1‐activated Smad3 govern Foxp3 expression.^35^ The analogous cooperative complex of Foxp3 with NFAT (nuclear factor of activated T cells) mediates Tregs function. Foxp3 mutations progressively disrupt its interaction with NFAT, thereby lacking suppressor function in a murine model of autoimmune diabetes.^36^ Smad3 cooperated with NFAT to improve histone acetylation in the enhancer region and induction of Foxp3. Consistent with these observations, SIS3（the Smad3 inhibitor） was capable of blocking Foxp3 expression.^37^ Smad4 is shown as the most universal coregulator of canonical TGF‐β signalling pathway. Nevertheless, it could be observed that there were significant differences between T cell‐specific Smad4^−/−^ mice and Smad2^−/−^ Smad3^−/−^ mice. TGF‐β‐mediated Foxp3 expression was completely reduced in CD4^+^ T cells from Smad2^‐/‐^Smad3^−/−^ mice, while Smad4 deficiency in CD4^+^ T cells resulted in the decrease in TGF‐β‐mediated Foxp3 expression.^34^ Thus, phosphorylated Smad2 and Smad3 cooperated with Smad4 to govern Foxp3 expression. In our study, the expression of Foxp3 was inhibited via down‐regulating the expression of Smad2 phosphorylation as well as Smad4, while the overexpression of Smad2 and Smad4 alleviated the ZIKV‐mediated down‐regulation of Foxp3.

Our study has limitations. For example, we have only demonstrated the reduced number of Tregs in ZIKV‐induced FGR mouse. It is unclear whether our conclusion can be extended to FGR caused by other conditions. Future study should establish additional FGR models to validate our findings, for example, in low calorie intake induced FGR in mice. Finally, our current results indicate that maternal immune tolerance mediated by Tregs plays a dominant role in FGR development. Targeting Tregs or its transcriptional factor Foxp3 might represent a novel strategy to reverse or treat early stage FGR.

## CONFLICTS OF INTEREST

The authors declared that they have no competing interests.

## AUTHOR CONTRIBUTIONS

YZX and WLG designed the study; YZX and MS collected the tissue samples; ZHW performed the mouse model; QQL and XYX collected clinical data and participated cells experiments and flow cytometric analysis; STG and WDP did Tregs isolation experiments and proliferation assay; YZX drafted the manuscript; and WLG supervised the study. All authors read and approved the final manuscript.

## Data Availability

All data generated or analysed during this study are included in this article.
